# Alpha-lipoic acid ameliorates the epithelial mesenchymal transition induced by unilateral ureteral obstruction in mice

**DOI:** 10.1038/srep46065

**Published:** 2017-04-05

**Authors:** Hyun Seop Cho, Jin Hyun Kim, Ha Nee Jang, Tae Won Lee, Myeong Hee Jung, Tae Ho Kim, Se-Ho Chang, Dong Jun Park

**Affiliations:** 1 Division of Nephrology, Department of Internal Medicine, Gyeongsang National University Hospital, Gyeongsang National University, Jinju, Gyeongnam, Republic of Korea; 2 Institute of Health Science, Gyeongsang National University School of Medicine, Jinju, Gyeongnam, Republic of Korea; 3 Biomedical Research Institute, Gyeongsang National University Hospital, Jinju, Gyeongnam, Republic of Korea; 4 Department of Internal Medicine, Changwon Gyeongsang National University Hospital and Gyeongsang National University School of Medicine, Changwon, Gyeongnam, Republic of Korea

## Abstract

The epithelial-to-mesenchymal transition (EMT) is one of mechanisms that induce renal interstitial fibrosis. Understanding EMT in renal fibrosis has important therapeutic implications for patients with kidney disease. Alpha-lipoic acid (ALA) is a natural compound with antioxidant properties. Studies for ALA are performed in acute kidney injury with renal tubular apoptosis, renal inflammation, and oxidative stress. We investigated the effects of ALA on EMT-mediated renal interstitial fibrosis in mice with unilateral ureteral obstruction (UUO). UUO mice developed severe tubular atrophy and tubulointerstitial fibrosis, with a robust EMT response and ECM deposition after 7 postoperative days. In contrast, ALA-treated UUO mice showed only moderate injury and minimal fibrosis and also larger reductions in the expression of ECM proteins, inflammatory factors, and EMT markers. ALA was shown to be involved in the suppression of infiltrating macrophages associated with EMT and the progression of interstitial fibrosis. It also lessened the destruction of the tubular basement membrane, by reducing the expression of matrix metalloproteinases. This is the first study to show that ALA modulates EMT in a UUO mouse model. Our results suggest that ALA merits further exploration as a therapeutic agent in the prevention and treatment of chronic kidney disease.

Tubulointerstitial disease is a common histopathologic feature of progressive renal disease of diverse causes^1^,^2^ and it strongly correlates with the deterioration of renal function. Inflammation of the tubulointerstitial compartment leads to fibrosis^3^,^4^ via excessive extracellular matrix (ECM) production, fibroblast activation/proliferation, monocyte and macrophage infiltration, and tubular atrophy^5^,^6^. Interstitial fibrosis is a hallmark of chronic renal failure and strongly correlates with the deterioration of renal function, regardless of the underlying disease.

Kidneys with ureteral obstruction develop progressive tubulointerstitial damage. Unilateral ureteral obstruction (UUO) is a well-characterized animal model of renal injury leading to tubulointerstitial fibrosis^7^,^8^. UUO in rats and mice produces the tubulointerstitial inflammation and fibrosis seen in humans with obstructive nephropathy^9^. Damage to the obstructed kidney is accompanied by tubular atrophy and progressive interstitial fibrosis, which reflect the excessive production and deposition of ECM in the interstitium^10^. ECM proteins, such as fibronectin and collagens, are secreted by myofibroblasts. These cells are derived from resident interstitial fibroblasts or from transformed epithelial cells, a process referred to as the epithelial-to-mesenchymal transition (EMT)^11^.

The progression of renal disease in UUO mice is associated with EMT, in which there is reciprocal up-regulation of fibroblast-specific protein-1 (FSP-1) and α-smooth muscle actin (α-SMA) expression and a decrease in E-cadherin expression^12^. With the loss of epithelial cell properties, myofibroblasts proliferate, migrate, and produce and deposit large amounts of ECM in the renal interstitium. The process is compounded by the infiltration of immune cells, particularly macrophages, which secret numerous pro-fibrotic factors^13^.

Alpha-lipoic acid (ALA) is a natural compound originally isolated from bovine liver. Its therapeutic action is based on its antioxidant properties. In humans, ALA is synthesized in the liver and other tissues with high metabolic activity, such as the heart and kidney. The utility of exogenous ALA in the treatment of diverse conditions, including diabetes, atherosclerosis, insulin resistance, neuropathy, neurodegenerative diseases, and ischemia-reperfusion injury, has been examined in clinical and experimental studies^14^,^15^,^16^. Dietary supplementation with ALA was shown to prevent the glomerular injury caused by diabetes mellitus^16^, to protect against drug-induced nephrotoxicity^17^, and to attenuate cisplatin-induced acute kidney injury (AKI)^18^,^19^.

Because EMT underlies the development of renal interstitial fibrosis and thus contributes to renal failure, therapeutic interventions aimed at its inhibition can prevent progressive kidney disease. As a candidate therapeutic agent, most studies of ALA have focused on its antioxidant properties, making use of the AKI model to investigate renal tubular apoptosis, renal inflammation, and oxidative stress. The aim of this study was to assess the anti-fibrotic effects of ALA in EMT-mediated renal interstitial fibrosis.

## Results

### ALA improves the histopathological changes in the kidneys given to UUO

**Figure 1:**
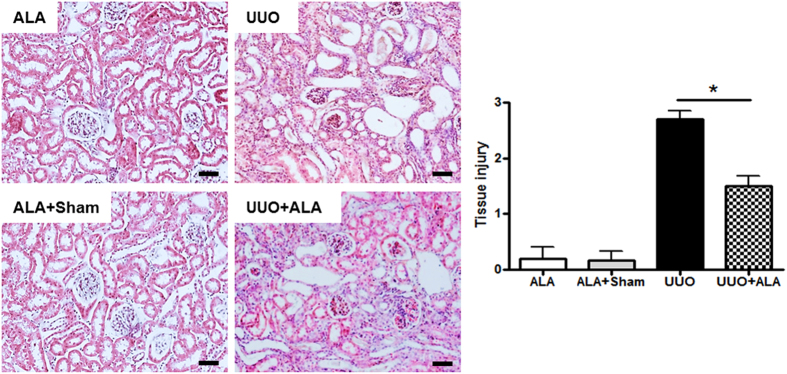
HE staining was performed to pathological changes at 7 days following UUO. Semiquantitative analysis showed result of tubulointerstitial injury in the kidneys. The scoring injury was described in the “Materials and Methods”. ALA; only ALA treated group, ALA + Sham; ALA treated and no ureteral ligated group, UUO; no ALA treated, but ureteral ligated group, UUO + ALA; ALA treated and ureteral ligated group. Values are expressed as means ± SE (*P < 0.05). Scale bar, 50 μm.

Histological examination by H&E staining showed normal renal cortex in ALA-treated (ALA) and ALA and sham-operated (ALA + Sham) kidneys. There were interstitial inflammatory cell infiltration, partial tubular expansion, a severe tubular atrophy, and swelling epithelial cells in the obstructed kidney (UUO, Fig. 1). The ALA + UUO group significantly exhibited an attenuated inflammatory cell infiltration, reduced tubular expansion and tubular atrophy, and less swelling epithelial cells as compared with UUO group.

### ALA ameliorates UUO-induced renal fibrosis

**Figure 2:**
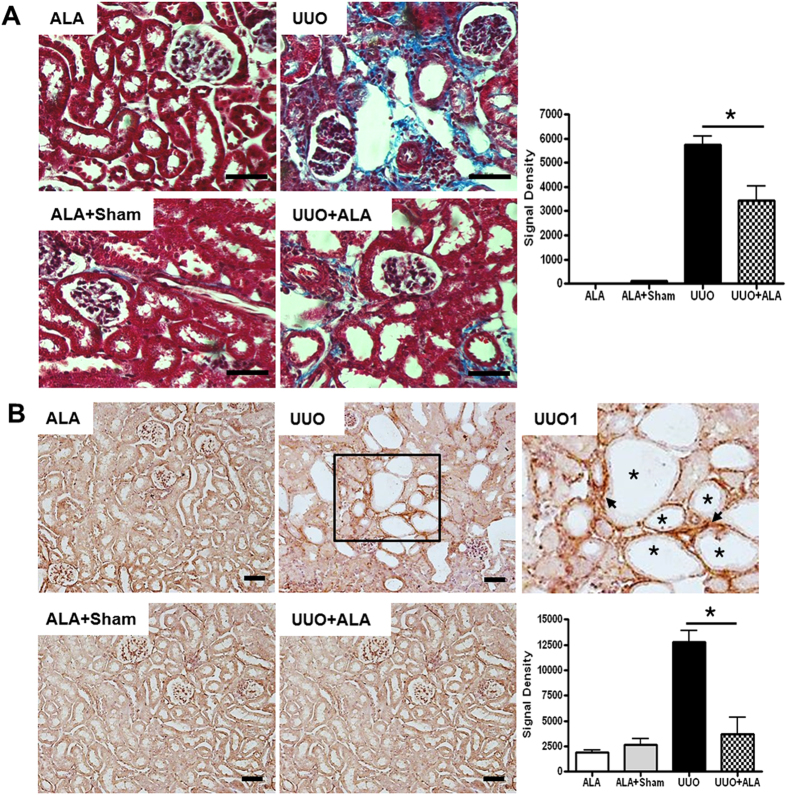
ALA ameliorates renal fibrosis. Masson’s trichrome staining (**A**) and immunohistochemical staining against collagen I (**B**) were performed. The severity of interstitial fibrosis and densitometric quantification was applied for collagen I positive areas were examined by densitometric quantification. ALA; only ALA treated group, ALA + Sham; ALA treated and no ureteral ligated group, UUO; no ALA treated, but ureteral ligated group, UUO + ALA; ALA treated and ureteral ligated group. Values are expressed as means ± SE (*P < 0.05). Scale bar, 50 μm. UUO1, enlarged image from insert in UUO. Arrow, tubular interstitial area. Asterisk, the dilated tubules.

Fibrosis examination of the Masson’s trichrome stained sections showed normal renal cortex in both ALA and ALA + sham kidneys. Obvious interstitial fibrosis was seen in the obstructed UUO kidneys, as shown by intense collagen deposition in the interstitium (blue staining in Fig. 2A). Semiquantitative scoring using an image analyzer confirmed that all UUO-only mice developed severe interstitial fibrosis, whereas in UUO + ALA mice only mild renal fibrosis was detected after 7 postoperative days (Fig. 2A). We then examined the expression of the ECM protein collagen I, a surrogate marker of renal fibrosis, in the kidney. Immunostaining with anti-collagen I antibody showed strong expression in the interstitium (arrow) adjacent to the dilated tubules (asterisk), with more intense staining in UUO mice than in UUO + ALA mice (Fig. 2B).

### UUO-induced EMT is ameliorated by ALA

**Figure 3:**
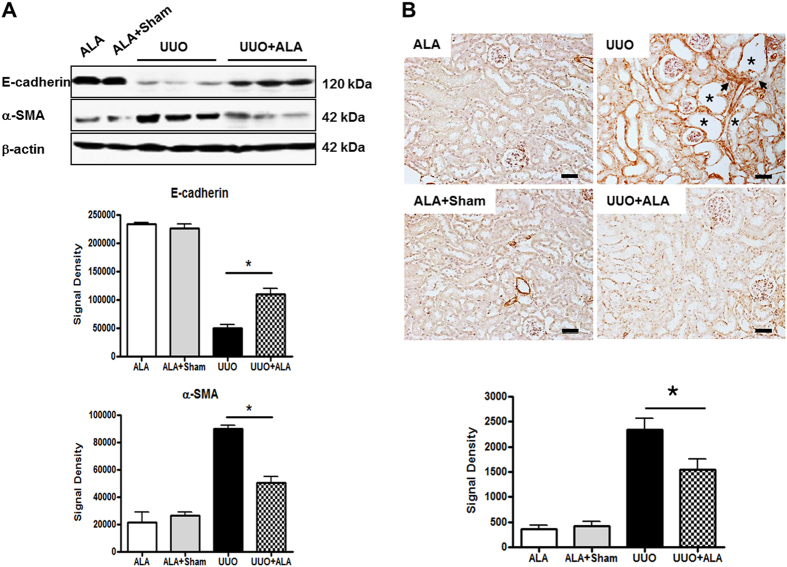
ALA decreases EMT by UUO. Immunoblot analysis was performed with a specific antibody against E-cadherin and *α*-SMA (**A**). Whole kidney were prepared and processed for immunoblot. *β*-actin was used as loading control and data were normalized against the density of *β*-actin by TotalLab TL100 v2006 software. Immunohistochemical staining was performed with a specific antibody against α-SMA (**B**). Scale bar, 50 μm. ALA; only ALA treated group, ALA + Sham; ALA treated and no ureteral ligated group, UUO; no ALA treated, but ureteral ligated group, UUO + ALA; ALA treated and ureteral ligated group. Values are expressed as means ± SE (*P < 0.05). Arrow, tubular interstitial area. Asterisk, the dilated tubules.

EMT, a key feature in the response to UUO, is characterized by a decrease in the cellular expression of intercellular epithelial adhesion molecules, such as E-cadherin, and the concomitant development of a mesenchymal phenotype, including the expression of α-SMA^20^. UUO led to a marked down-regulation of E-cadherin and the up-regulation of α-SMA in the obstructed kidneys, whereas ALA treatment moderately ameliorated these effects (Fig. 3A). There was widespread expression of α-SMA in in the interstitium (arrow) adjacent to the dilated tubules (asterisk) of the obstructed kidneys, as well as strong staining of intrinsic interstitial fibroblasts. By contrast, in ALA + UUO mice, α-SMA-positive signals were significantly decreased (1.5-fold, UUO vs. ALA + UUO; *P* < 0.05) (Fig. 3B).

### ALA decreases UUO-induced TGF-β1 and pSmad expression

**Figure 4:**
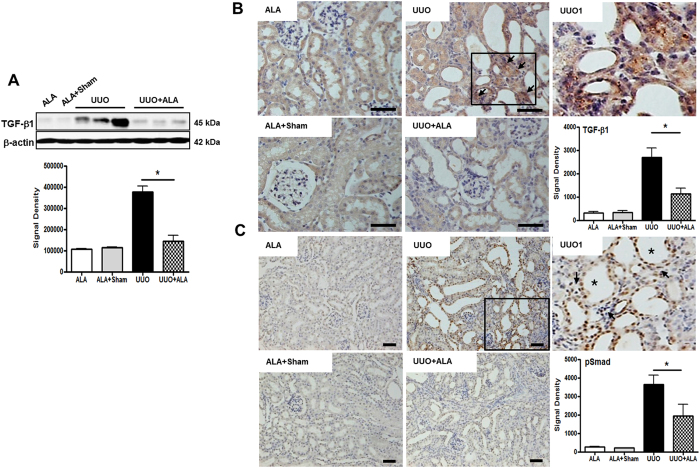
ALA ameliorates expression of TGF-*β*1 and pSmad. Immunoblot analysis was performed with a specific antibody against TGF-*β*1 (**A**). *β*-actin was used as loading control and data were normalized against the density of *β*-action by TotalLab TL100 v2006 software. Immunohistochemical staining was performed with a specific antibody against TGF-*β*1 (**B**) and pSmad (**C**). The TGF-*β*1 expression was predominantly in the interstitium of UUO kidneys (arrow). Densitometric quantification for TGF-*β*1 and pSmad was applied to each group. Scale bar; 50 μm. ALA; only ALA treated group, ALA + Sham; ALA treated and no ureteral ligated group, UUO; no ALA treated, but ureteral ligated group, UUO + ALA; ALA treated and ureteral ligated group. Values are expressed as means ± SE (*P < 0.05). UUO1, enlarged image from insert in UUO. Arrow, tubular interstitial area. Asterisk, the dilated tubules.

EMT is induced by a wide variety of stimuli in the setting of chronic kidney disease (CKD)^21^. TGF-β is a potent pro-fibrotic factor that promotes EMT by directly stimulating the expression of ECM proteins in renal cells^22^. In addition, it enhances the recruitment of inflammatory cells, especially macrophages. Western blot analyses of total kidney lysates showed a robust induction of TGF-β1 in UUO mice but only a minor response in ALA + UUO mice (Fig. 4A). Specifically, TGF-β1 expression was significantly higher in the interstitium of UUO kidneys than in ALA + UUO kidneys (2.38-fold; *P* < 0.05; Fig. 4B). Immunohistochemistry studies showed TGF-β1 staining predominantly in the renal interstitium of UUO mice (arrow, Fig. 4B) whereas its expression in the renal tubules of the ALA and ALA + sham mice was negligible. EMT induction by TGF-β1 decreased the E-cadherin expression associated with the nuclear translocation of phosphorylated Smad (pSmad)^23^. pSmad-positive signals were mainly detected in the nuclei of dilated tubular epithelial cells and in a few interstitium (arrow) adjacent to the dilated tubules (asterisk) and were significantly higher in the UUO group than in the other groups (Fig. 4C).

### ALA suppresses UUO-induced NF-κB pathway activation, mononuclear cell infiltration, and ICAM-1 protein expression

**Figure 5:**
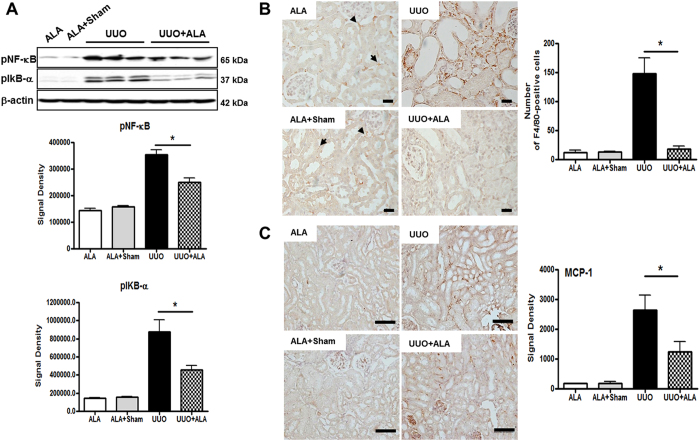
ALA reduces renal fibrosis by regulating inflammation. Immunoblot analysis was performed with a specific antibody against phosphorylated NF-κB and phosphorylated IκB-*α*. Data were normalized against the density of *β*-action by TotalLab TL100 v2006 software (**A**). Immunohistochemical staining was performed with a specific antibody against F4/80. A few F4/80-positive cells were observed in the peritubular areas (arrow) and interstitial foci (arrowhead) of ALA and ALA + sham kidneys (**B**) and MCP-1 (**C**). Scale bar; 100 μm. ALA; only ALA treated group, ALA + Sham; ALA treated and no ureteral ligated group, UUO; no ALA treated, but ureteral ligated group, UUO + ALA; ALA treated and ureteral ligated group. Values are expressed as means ± SE (*P < 0.05).

There is increasing evidence of a role for nuclear factor κB (NF-κB) activation in the initial stages of EMT^24^. Because ALA was previously shown to suppress the NF-*κ*B pathway^25^,^26^,^27^, we examined whether the NF-κB pathway was sensitive to ALA. The results showed that the levels of phosphorylated NF-κB and phosphorylated IκB-α, components of the NF-κB signaling pathway, were induced by UUO and attenuated by ALA in UUO mice (Fig. 5A). Changes in the expression of these phosphorylated proteins were correlated with the ALA-stimulated expression of E-cadherin and α-SMA. Mononuclear cell infiltration is an additional measure of progressive renal injury. ALA is known to suppress the NF-κB-dependent up-regulation of monocyte chemoattractant protein 1 (MCP-1), a chemokine promoting macrophage infiltration, and of ICAM-1 *in vivo* and *in vitro*^26^. It also attenuated the cisplatin-induced increase in the phosphorylation and nuclear translocation of the NF-κB p65 subunit in kidney tissue^27^. In the current study, a few F4/80-positive cells were observed in the peritubular areas (arrowhead) and interstitial foci (arrow) of ALA and ALA + sham kidneys but the difference was not statistically significant (Fig. 5B). However, significant amounts of interstitial cellular infiltration were present in the interstitium of UUO kidneys, whereas in the ALA + UUO kidneys the cells were mainly in peritubular areas and there were significantly fewer macrophages (18 vs. 148 in UUO kidneys; *P* < 0.05; Fig. 5B). In the obstructed kidneys, MCP-1 expression was highly induced whereas ALA treatment caused a dramatic reduction in MCP-1 levels (Fig. 5C).

### ALA ameliorates the UUO-induced expression of MMP-2 and 9

**Figure 6:**
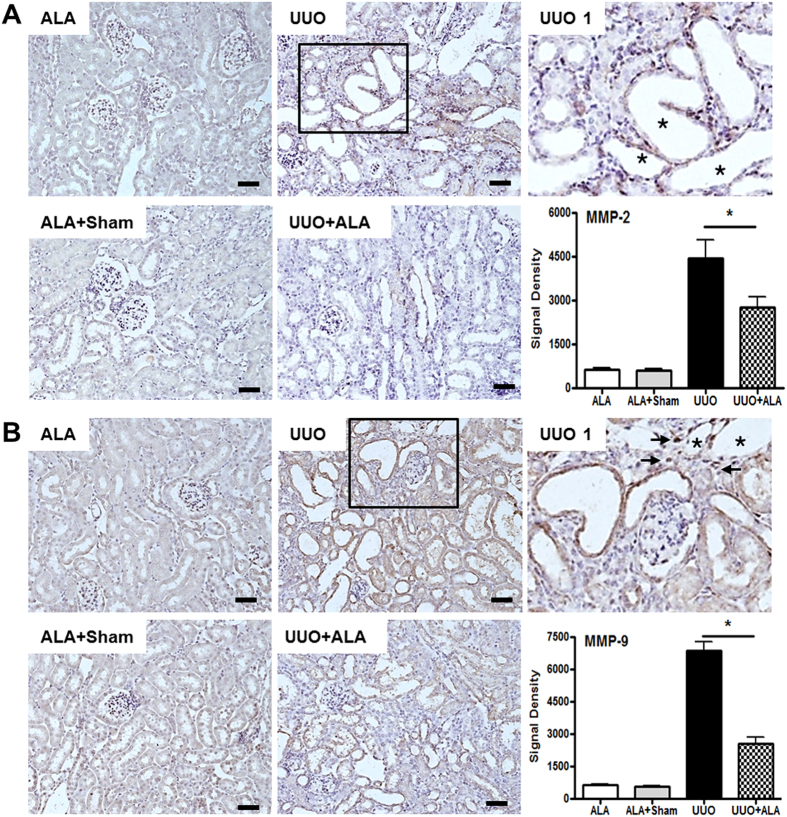
ALA decreases the expression of MMP-2 and MMP-9. Immunohistochemical staining was performed with a specific antibody against MMP-2 (**A**) and MMP-9 (**B**). Densitometric quantification for MMP-2 and MMP-9 was applied to each group. ALA; only ALA treated group, ALA + Sham; ALA treated and no ureteral ligated group, UUO; no ALA treated, but ureteral ligated group, UUO + ALA; ALA treated and ureteral ligated group. Values are expressed as means ± SE (*P < 0.05). Scale bar; 50 μm. Asterisk, the dilated tubules. UUO1, enlarged image from insert in UUO. Arrow, tubular interstitial area. Asterisk, the dilated tubules.

During EMT in the kidney, the integrity of the tubular basement membrane (TBM) is gradually destroyed, in a process that includes the up-regulation of matrix proteinases. Both MMP-2 and MMP-9 participate in EMT-mediated obstructive nephropathy^28^,^29^. ALA inhibited MMP-9 expression in smooth muscle cells from the thoracic aorta^25^. In the UUO kidneys, both MMP-2 and MMP-9 were expressed in the epithelial cells of the dilated tubules (asterisk) (Fig. 6A and B) and occasionally in interstitial areas adjacent to the dilated tubules (arrow) (Fig. 6B) whereas their expression was reduced by ALA (Fig. 6).

### The contralateral kidney has normal morphology and histology

The pathology in the contralateral kidney was similar to that of ALA and ALA + Sham groups (Supplement Figure 1). The contralateral kidney 7 days after UUO operation showed normal architecture and histology.

## Discussion

The severe tubular atrophy and tubulointerstitial fibrosis that developed in the UUO mice included a robust EMT and the deposition of large amounts of ECM. However, in UUO mice treated with ALA, only moderate, histologically identifiable renal injury and minimal fibrosis were observed. At the molecular level, the expression of ECM proteins, fibrogenic and inflammatory factors (TGF-β1 and MCP-1), and EMT markers (E-cadherin, α-SMA, and pSmad) was much lower in ALA-treated than in non-treated UUO mice. This is the first demonstration that ALA modulates EMT in a UUO mouse model of the fibrotic, obstructed kidney.

EMT is a major pathway that leads to fibrosis in the kidney^30^. In humans with fibrotic kidneys, strong mesenchymal marker expression is accompanied by the deposition of collagen I among the renal tubules and massive interstitial fibrosis in the renal cortex^31^. Studies showed that, in UUO mice, fibroblasts and myofibroblasts (identified based on the markers FSP-1 and α-SMA, respectively) are increased after 7 days, indicating EMT activation^32^,^33^. Decreased E-cad and increased α-SMA expression are typical EMT features. In this study, ALA suppressed UUO-induced tubular interstitial fibrosis through ameliorating down-regulation of E-cad and the up-regulation of α-SMA expression.

Ureteral obstruction causes to up-regulation of MCP-1 and TGF-β^7^,^34^. TGF-β recruits monocytes (including F4/80-positive interstitial macrophages), induces EMT, and stimulates collagen production^35^. TGF-β is a major contributor to the cellular process that causes renal tubular epithelial cells to become ECM-producing myofibroblasts^20^,^23^,^36^. TGF-β1 binds to its type II receptor and then forms a complex with ALK5, which leads to activation of the intracellular signaling pathway mediated by Smad proteins^37^,^38^,^39^,^40^. The induction of EMT by recombinant human TGF-β1 decreased E-cadherin expression in association with the nuclear translocation of phosphorylated Smad2 and 3^23^. In this study, we found that ALA significantly suppressed TGF-β1 expression in the interstitium and pSmad expression in the tubular epithelial cells and in a few interstitial cells adjacent to damaged tubules of the obstructed kidneys.

Macrophages are a key source of the cytokines associated with fibrosis^7^,^41^. Their interaction with proximal tubular epithelial cells leads to interstitial fibrosis in the injured kidney. Macrophage infiltration is preceded by the local expression of chemokines, such as the proinflammatory cytokine MCP-1, chemokine receptors, and adhesion molecules^8^. In a murine model, macrophage depletion markedly reduced myofibroblast formation and interstitial fibrosis in the kidney^42^. Our data demonstrated that ALA is able to suppress macrophage infiltration and therefore EMT and the progression of interstitial fibrosis.

In the kidney, the obvious initial change of EMT is the destruction of TBM integrity, attributed to proteolysis mediated by MMPs^28^,^29^. MMP-2 causes structural alterations in the TBM that promote tubular atrophy, fibrosis, and renal failure. These changes were observed in transgenic mice with renal proximal tubular epithelial expression of active MMP-2^28^. Conversely, in the kidneys of mice genetically engineered with a null mutation of the endogenous MMP-9 gene, interstitial fibrotic lesions did not develop. Similarly, our data also showed that ALA ameliorated upregulated MMP-2 and MMP-9 expression in the tubular epithelial cells and in some interstitial cells adjacent to damaged tubules of the obstructed kidney. Reduced expression of MMP-2 and MMP-9 by ALA well correlated with decreased EMT features as well as less interstitial fibrosis in the obstructed kidney.

Most of studies in kidney injury as well as other tissue damage are focused on antioxidant properties for ALA^14^,^15^,^16^. However, we tried to find another ALA’s role in this study besides antioxidant properties and our data provide the anti-fibrotic effects of ALA in EMT-mediated renal interstitial fibrosis. There was one limitation to this study. Renal functional study can be an important part of several studies, especially in AKI model. Because we focused on histological and molecular changes of UUO kidney, we initially did not check the biochemical data such as BUN and serum creatinine level. However, many previous studies have reported that BUN and serum creatinine level was not significantly affected by only ipsilateral UUO due to the presence of a contralateral kidney with good renal function^43^,^44^,^45^,^46^,^47^,^48^. Practically, we confirmed normal morphology and histology of contralateral kidney. This may suggest that BUN and serum creatinine level are not good markers of renal function in an UUO animal model. Even if the changes biochemical data on renal function exist in our study, we don’t think that it would not affect histological changes of UUO kidney.

We found that ALA suppressed UUO-induced tubular interstitial fibrosis through ameliorating the EMT. The plausible mechanisms might be as follows: (1) ALA preferentially inhibits infiltration of mononuclear inflammatory cells expressing F4/80 into the interstitium resulting in mitigating the expression of TGF-β1 released from these cells in the interstitium of the obstructed kidneys. These anti-inflammatory properties of ALA was also identified by reduction of NF-κB and MCP-1 expression; (2) MMP-2 and MMP-9 expression, which are induced by TGF-β1, in the tubular epithelial cells and in the interstitial cells adjacent to damaged tubules of the obstructed kidney was reduced by direct and/or indirect ways through ALA administration. The upregulated MMP-2 and MMP-9 expression in the obstructed kidney might be responsible for degradation of basement membrane finally leading to the tubular epithelial cells dissociation. Areas of basement membrane with dissociated epithelial cells have a mesenchymal phenotype invading the interstitium. Disruption of epithelial cells by proteases like MMP-2 and MMP-9 is sufficient to trigger EMT featured with decreased E-cad and increased α-SMA expression. This expression of MMP-2 and MMP-9 is also positively correlated with the degree of interstitial fibrosis, the byproduct of EMT, in the obstructed kidney. But changes including mild or less interstitial fibrosis were detected in ALA-treated obstructed kidneys.

A better understanding of the mechanism by which EMT contributes to the development of CKD will aid in the development of therapeutic interventions in patients with kidney disease. The current study indicates that ALA might be used as a therapeutic agent in the prevention and treatment of CKD.

## Methods

### Animals, surgery, and tissue preparation

Male C57BL/6 mice (10 weeks of age) were maintained in a 12 h light/dark cycle in a temperature- and humidity-controlled facility. Standard mice chow and water were provided ad libitum. The animal experiments were reviewed and approved by the Gyeongsang National University Guide for the Care and Use of Laboratory Animals (Approved ID: GNU-130813-M0056). The 28 mice were divided into four groups: ALA (50 mg/kg, i.p., *n* = 7), ALA + Sham (*n* = λ, UUO (*n* = 7), and ALA + UUO (*n* = 7). ALA injection is started 24 hour before the UUO and continued afterward for 7 days. Mice undergoing UUO surgery were anesthetized with inhaled isoflurane (2.5%). In the UUO group, a left flank incision was made in the anesthetized mice, after which the left ureter was ligated completely at the ureteropelvic junction using double silk sutures. The right kidney was also exposed, but the ureter was not ligated. A left flank incision was also made in mice in the sham group, after which the kidney was exposed but the ureter was not ligated. The study was performed strictly according to the guidelines developed by the Gyeongsang National University Guide for the Care and Use of Laboratory Animals. All efforts were made to minimize suffering. All of the animals were placed on regular diets, allowed free access to tap water, and euthanized at day 7 post-UUO/sham surgery. This time point was chosen because it is sufficient to observe the development of tubular and interstitial cell apoptosis, as demonstrated in previous studies^43^. At the time of euthanization, both kidneys were removed and tissue samples were either fixed (10% buffered formaldehyde solution) for histopathologic studies or snap-frozen in liquid nitrogen for western analysis.

### Renal pathology

Kidneys were routinely fixed in 4% phosphate-buffered paraformaldehyde and paraffin embedded. Tissue sections at 5 μm were obtained. Paraffin wax was removed with xylene, and sections were rehydrated with ethanol. After washing, the sections were stained with H&E and Masson trichrome. H&E staining was for histopathological analysis. MT staining was to assess the tissue fibrotic changes as well collagen-1 immunohistochenical staining. The semi-quantitative scoring for H&E staining were examined on the degree of interstitial injury that assigned points (0 to 3) for the extent of interstitial fibrosis, tubular atrophy (defined as luminal dilation and flattened tubular epithelial cells) and interstitial inflammatory cell infiltration. Tissue injury (interstitial fibrosis, tubular atrophy, and interstitial inflammatory cell infiltration) has been scored by grading the percentage of affected under a high-powered field ( × 400) with minor modifications from previous study^49^; 0, 0%; 1, <30%; 2, 31 to 60%; 3, 61 to 100% and all scorings were summed and represented as average values on the graph. Ten tubulointerstitial fields that were randomly selected were assessed in each section, and the density of trichrome-positive signals was analyzed using NIS-Elements BR 3.2 (Nikon, Japan).

### Protein preparation and western blot

The previously removed kidneys were extracted by homogenization in lysis buffer [1 × PBS (pH 7.4) with 1% Triton X-100 and 1 mM EDTA] containing 10 μM leupeptin and 200 μM PMSF. Protein concentrations were determined using a protein assay kit (Bio-Rad, Hercules, CA, USA), with BSA as the standard. Thirty micrograms of total protein was applied to a 10–12% SDS–polyacrylamide gel. After electrophoresis, the proteins in the gel were transferred to a nitrocellulose membrane (Schleicher & Schuell, Dassel, Germany). The blots were probed with primary antibodies to polyclonal anti-TGF-β1 (diluted 1:500; sc146, Santa Cruz Biotechnology, Santa Cruz, CA, USA) and monoclonal anti- E-cadherin (diluted 1:2000; 610181, BD bioscience, San Jose, CA, USA), anti-α-SMA (diluted 1:500; A5228, Sigma, St. Louis, MO, USA), anti-β-actin (diluted 1:500; 3033, Cell Signaling Technology, Danvers, MA, USA), anti-pNF-kB (diluted 1:5000; A5441, Sigma, St. Louis, MO, USA), and anti-p-IκBα (diluted 1:1000; sc8404, Santa Cruz Biotechnology, Santa Cruz, CA, USA) at 4 °C overnight. The blots were then incubated with secondary antibody. Reactivity was visualized using the ECL kit. The β-actin antibody (Sigma) served as the loading control. The densitometric analysis was performed for quantitative analysis of all data. Immunoblot were performed for the expression levels of EMT-related factors (E-cad, α-SMA, TGF-β1) and the expression levels of inflammation-related factors (pNF-kB, p-IκBα).

### Immunohistochemistry

An avidin-biotinylated-HRP (ABC; Vector Laboratories, Burlingame, CA, USA) kit was used for immunohistochemistry studies, together with 5 μm-thick paraformaldehyde-fixed, paraffin-embedded kidney sections. After their incubation with 1% normal serum, the sections were treated with each primary antibodies to polyclonal anti-Collagen-1 (diluted 1:30; 234167, Calbiochem, Menlo Park, CA, USA), anti-TGF-β1 (diluted 1:100; sc146, Santa Cruz Biotechnology, Santa Cruz, CA, USA), anti-pSmad (diluted 1:100; SC11769-R, Santa Cruz Biotechnology, Santa Cruz, CA, USA), anti-F4/80 (diluted 1:100; 14-4801, ebioscience, San Diego, CA, USA), anti-MCP-1 (diluted 1:100; sc1785, Santa Cruz Biotechnology, Santa Cruz, CA, USA), anti-MMP-2 (diluted 1:50; sc10736, Santa Cruz Biotechnology, Santa Cruz, CA, USA), anti-MMP-9 (diluted 1:100; sc19016, Santa Cruz Biotechnology, Santa Cruz, CA, USA) and monoclonal anti-α-SMA (diluted 1:500; A5228, Sigma, St. Louis, MO, USA) at 4 °C for 16 h. They were then washed in PBS (pH 7.4), incubated for 90 min with secondary antibody, and then with ABC for 60 min at room temperature. After rinsing the sections with PBS, the reactions were developed using 0.027% 3,3-diaminobenzidine tetrahydrochloride (Sigma, St. Louis, MO, USA) with 0.003% H_2_O_2_. The sections were counterstained with hematoxylin to visualize the cell nuclei. Immunohistochemistry were for the expression levels of EMT-related factors (α-SMA, TGF-β1, pSmad) and the expression levels of inflammation -related factors (F4/80, MCP-1, MMP-2, MMP-9). Ten fields that were randomly selected were assessed in each section, and the density of target signals was analyzed using NIS-Elements BR 3.2 (Nikon, *Japan*). The densitometric analysis was performed for quantitative analysis of all data.

### Statistical analysis

Statistical analysis was conducted using Sigma Plot 7.0. Statistical differences between the experimental groups were determined using analyses of variance and Student’s *t*-tests. P < 0.05 was considered statistically significant. Values are represented as the mean ± the standard error of the mean.

## Additional Information

**How to cite this article:** Cho, H. S. *et al*. Alpha-lipoic acid ameliorates the epithelial mesenchymal transition induced by unilateral ureteral obstruction in mice. *Sci. Rep.* **7**, 46065; doi: 10.1038/srep46065 (2017).

**Publisher's note:** Springer Nature remains neutral with regard to jurisdictional claims in published maps and institutional affiliations.

## Supplementary Material

Supplementary Information
