# Supplementary material for: Alpha-lipoic acid ameliorates the epithelial mesenchymal transition induced by unilateral ureteral obstruction in mice
Source: Sci Rep. 2017 Apr 5;7:46065. doi: 10.1038/srep46065 (PMC5380949; doi:10.1038/srep46065)
Supplement: Supplementary Information [file srep46065-s1.pdf]

Supplementary Information

Alpha-lipoic acid ameliorates the epithelial mesenchymal transition induced by unilateral ureteral obstruction in mice

Hyun Seop Cho[1§], Jin Hyun Kim[2,3§], Ha Nee Jang[1], Tae Won Lee[1], Myeong Hee Jung[3], Tae Ho Kim[3], Se-Ho Chang[1,2], Dong Jun Park[2,4]*

[1]Division of Nephrology, Department of Internal Medicine, Gyeongsang National University Hospital, Gyeongsang National University, Jinju, Gyeongnam, Republic of Korea

[2]Institute of Health Science, Gyeongsang National University School of Medicine, Jinju, Gyeongnam, Republic of Korea

[3]Biomedical Research Institute, Gyeongsang National University Hospital, Jinju, Gyeongnam, Republic of Korea

[4]Department of Internal Medicine, Changwon Gyeongsang National University Hospital and Gyeongsang National University School of Medicine, Changwon, Gyeongnam, Republic of Korea

*Author for correspondence: Dong Jun Park, MD, PhD

Department of Internal Medicine, Changwon Gyeongsang National University Hospital and Gyeongsang National University School of Medicine, Samjeongja-ro 11, Seongsan-gu, Changwon-si, Gyeongsangnam-do, Republic of Korea.

Tel: +82-55-214-3735; Fax: +82-55-214-3250; E-mail: drpdj@naver.com

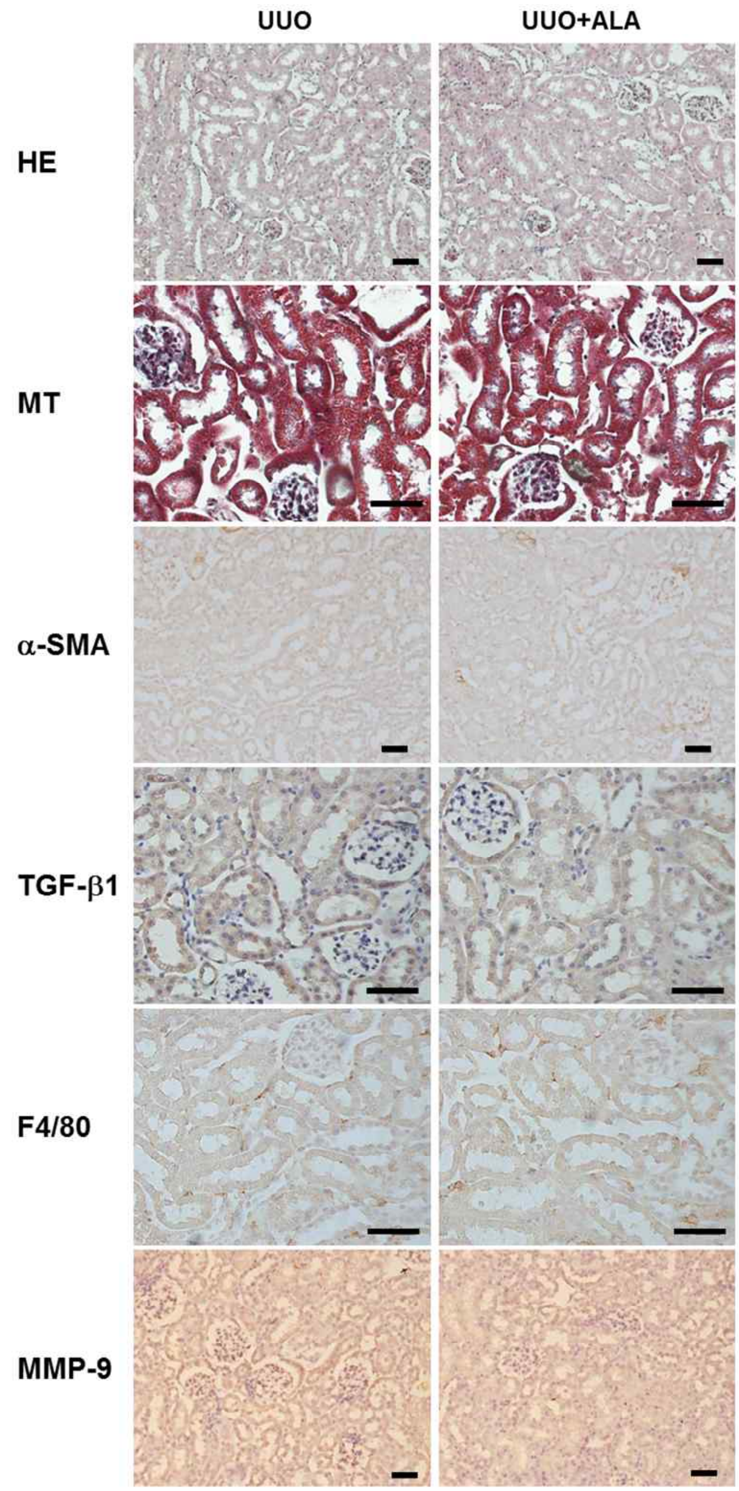

Supplementary Figure S1. The representative histology of contralateral kidney in each group. HE, Masson's trichrome (MT) staining, α-SMA, TGF-β1, F4/80, and MMP-9 were performed in the sections of contralateral kidneys 7 days after UUO. UUO; no ALA treated, but ureteral ligated group, UUO+ALA; ALA treated and ureteral ligated group. Scale bar, 100 μm.
